# Trauma by the Numbers: A Cross-Sectional Analysis and Categorization of Trauma Cases in the Gaza War

**DOI:** 10.3389/ijph.2025.1607877

**Published:** 2025-05-22

**Authors:** Muaaz Wajahath, Elias Nasser, Tariq Nayfeh, Bilal Irfan, Rohit Balasundaram, Mosab Nasser, Khaled J. Saleh

**Affiliations:** ^1^ Michigan State University College of Human Medicine, Lansing, MI, United States; ^2^ FAJR Scientific, Houston, TX, United States; ^3^ The University of Texas Southwestern Medical Center, Dallas, TX, United States; ^4^ Center for Bioethics, Harvard Medical School, Boston, MA, United States; ^5^ Center for Surgery and Public Health, Brigham and Women’s Hospital, Boston, MA, United States; ^6^ Department of Neurology, University of Michigan Medical School, Ann Arbor, MI, United States; ^7^ Wayne State University School of Medicine, Detroit, MI, United States

**Keywords:** trauma epidemiology, conflict zones, Gaza war, injury classification, humanitarian efforts

## Abstract

**Objectives:**

To categorize and analyze trauma cases from the Gaza war, identifying injury patterns and informing future humanitarian efforts in conflict zones.

**Methods:**

A cross-sectional study was conducted in April 2024 at European Gaza Hospital. Data on demographics and injury types were collected from patients treated by FAJR Scientific’s surgical team. Injuries were classified into primary (directly conflict-related), secondary (indirectly conflict-related), and tertiary (unrelated to the conflict). Statistical analyses included Kruskal-Wallis H test, logistic regression, and Fisher’s Exact Test.

**Results:**

Among the 80 surgical cases analyzed, primary injuries were the most common (53%), predominantly affecting males aged 30–39. Secondary injuries accounted for 14% of cases, while tertiary injuries represented 33%. Significant associations were found between age categories and injury classifications (p < 0.05).

**Conclusion:**

The prevalence of primary injuries highlights the severe impact of conflict on civilians, particularly middle-aged males. The findings suggest the need for enhanced orthopedic surgical capacity, integrated chronic disease management, and specialized pediatric care in conflict zones. Improved data collection and analysis are essential for optimizing medical interventions and resource allocation.

## Introduction

Understanding trauma epidemiology in conflict zones is crucial to guide effective medical interventions and resource allocation [1–3]. This information is particularly relevant in acute crisis settings impacted by sustained damage to civilian and hospital infrastructure, as seen in the Gaza war (2023-ongoing) [4–7]. This study aims to categorize and analyze trauma cases from the ongoing conflict, which has resulted in substantial casualties and significant damage to Gaza’s healthcare infrastructure [8, 9].

In many conflict zones, registries are not available to track contributions to trauma care by non-governmental organizations (NGOs) and other humanitarian entities [2, 10, 11]. The primary objective of this research is to systematically categorize trauma cases treated during the conflict, utilizing data collected by FAJR Scientific (hereafter FAJR), a U.S.-based nonprofit organization dedicated to global surgical care. This study not only categorizes trauma cases but also aims to identify patterns that can inform future humanitarian efforts in similar contexts. Injuries are classified into three categories: primary (directly conflict-related), secondary (indirectly conflict-related), and tertiary (unrelated to the conflict).

The FAJR Surgical Team collected data at the European Gaza Hospital (EGH) in Khan Yunis in April 2024, including demographic history, injury types, and treatment outcomes. Data were gathered on all treated cases, with over eighty surgeries performed at EGH across a period of 10 days. This dataset has thereafter been subject to a categorization and analysis of trauma cases, by focusing on the period immediately prior to the Israeli military ground offensive in neighboring Rafah.

Beyond the categorization of trauma cases, this study secondarily aims to examine the frequency of cases based on classification, explore potential causal factors, and future considerations for treatment and care. By identifying the most frequent cases and surgeries, this study aims to establish a correlative link between primary, secondary, and tertiary cases. This correlation is vital for informing the composition of trauma medical teams and guiding medical aid procurement, not only in the context of the Gaza war but future conflicts [12].

The crisis in Gaza in particular has brought a renewed focus of the global medical community on the importance of understanding trauma epidemiology in conflict-affected regions [13]. Intense military operations and displacement of civilian populations can lead to a deterioration in health prevention and outcomes; these challenges are compounded by the barriers that Palestinian health systems face in consistently gathering comprehensive data due to telecommunications blackout, cyberattacks, and destruction of medical infrastructure [14–16]. When registries are incomplete or non-existent, NGOs and humanitarian actors become vital conduits for both patient care and data collection [17]. In doing such, their role extends far beyond offering direct clinical services—by systematically recording details on patient demographics, mechanisms of injury, and clinical outcomes, these groups fill an essential gap in public health surveillance [18].

Public health experts increasingly recognize that trauma extends beyond the immediate surgical procedures [19, 20]. High rates of blast injuries, shrapnel wounds, and fractures translate into long-term disability, psychological distress, and chronic disease exacerbation, all of which affect population health long-term in a multitude of ways [21, 22]. Careful documentation of these injuries can reveal demographic patterns, identification of particularly vulnerable groups, and exposes gaps in current approaches to emergency response [23, 24]. Findings from such analyses can also shape future efforts to deliver specialized care, during or after acute periods of warfare, strengthen supply chains for surgical materials (subject to political constraints), and implement sustainable rehabilitation services for those who survive acute injuries but continue to bear substantial levels of morbidity [25].

## Methods

### Study Design

This cross-sectional study was designed to categorize and analyze trauma cases resulting from the Gaza war over a two-week period in April 2024.

### Setting

The study was conducted during an emergency medical mission led by FAJR in April 2024. This mission took place at the European Hospital in the Al-Fukhari neighborhood of the Gaza Strip’s Khan Yunis governorate. FAJR coordinated this mission with a multidisciplinary team of 16 physicians and surgeons from the United States and the United Kingdom, as well as international governmental and nongovernmental assistance. The mission aimed to provide surgical interventions and gather comprehensive patient data to inform this study and future interventions. The duration of the mission was 16 days, during which over 500 patients were examined and more than 80 surgeries were performed.

### Data Collection

Comprehensive data collection included patient demographics (age), injury specifics (type, location, mechanism of injury, severity), and treatment details (surgical procedures performed and outcomes) from patient records collected by the FAJR team. This extensive dataset provided a robust foundation for subsequent analysis. While over 500 patients were examined, only 80 patients received surgical intervention by members of the FAJR, reducing the total number of patients considered in this study. The inclusion criteria for the study were: patients of any age and gender who sustained trauma and were treated at EGH by FAJR personnel during the specified mission period. Exclusion criteria included uninjured patients and those treated outside the specified mission period.

Injuries were retrospectively categorized based on criteria developed by FAJR Scientific for optimizing public health surveillance by combining strands of existing protocols on blast injuries and conflict-related injuries. Assessments of classification were made a team of authors and clinicians, with any disagreements resolved by additional authors:

1. Primary Injuries: Direct conflict-related injuries resulting from explosive devices, high-velocity projectiles, and shrapnel. These included traumatic amputations, open fractures, and severe soft tissue damage requiring immediate surgical intervention.
2. Secondary Injuries: Indirect conflict-related injuries sustained as a consequence of the conflict environment. This category encompassed injuries such as crush syndromes, fractures, and dislocations resulting from structural collapses, and traumatic asphyxia from crowd crushes.
3. Tertiary Injuries: Non-conflict-related injuries that were coincidentally treated during the conflict period. These included degenerative joint diseases, chronic illnesses, and common injuries or presentations.

### Ethical Considerations

Ethical approval was obtained from relevant committees, including the Palestinian Ministry of Health and FAJR’s Ethics Committee. Informed consent was obtained from all patients or their surrogate decision-makers. Confidentiality and anonymity of patient data were strictly maintained, with data stored in secure systems.

### Statistical Analysis

Initial attempts to use ANOVA for evaluating differences in age distribution across case classifications were unsuccessful due to violations of normality and homogeneity of variances assumptions, which was confirmed by conducting a Shapiro Wiween case classification and age. Binary logistic regression categorized case classifications and Levene’s test was used for homogeneity. Consequently, the Kruskal-Wallis H test, a non-parametric alternative, was employed to compare age distributions across different case classifications. Further analysis involved logistic regression to examine associations between two groups: primary and non-primary, while multinomial logistic regression retained the original three categories: primary, secondary, and tertiary. To examine associations between age categories and case classifications, Fisher’s Exact Test with Monte Carlo simulation was utilized, given the small sample sizes and presence of zero counts. This robust method provided accurate p-value estimates, confirming significant associations between age categories and case classifications despite the data limitations. Statistical analyses were performed using Python, ensuring rigorous evaluation of the dataset.

## Results

### Demographic Analysis

**FIGURE 1:**
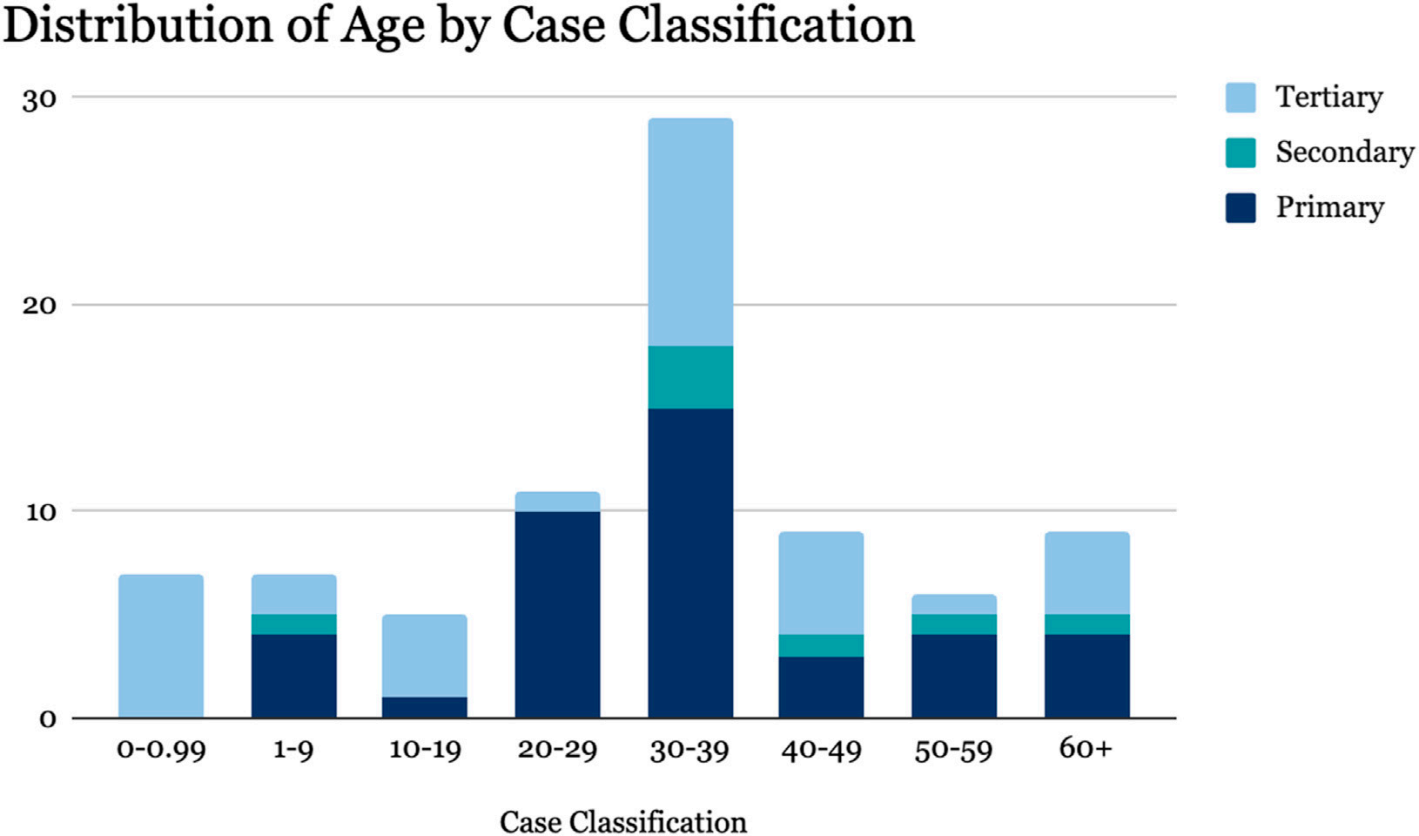
Demographics and age distribution by injury classification (Palestine, 2024).

A comprehensive analysis was conducted on trauma cases categorized into primary, secondary, and tertiary injury classifications utilizing the aforementioned classification guidelines (Figure 1). The age distribution of patients is summarized in Table 1. Among primary injuries, the highest incidence was in the 30–39 age group (37%) followed by the 20–29 age group (24%). Secondary injuries were most frequent in the 30–39 age group (43%). Tertiary injuries were predominantly observed in the less than 1-year-old age group (20%) and the 30–39 age group (31%). Due to the violations of ANOVA assumptions, the Kruskal-Wallis H test was employed, which revealed a Chi-Square statistic of 1.9308 with 2 degrees of freedom and a p-value of 0.3808, indicating no significant difference in age distributions among case classifications. Logistic regression analyses also examined the relationship between age and case classifications. Binary logistic regression results showed a weak positive, non-significant association between age and the likelihood of a case being classified as primary. Multinomial logistic regression confirmed weak, non-significant associations across all categories. Fisher’s Exact Test, supported by Monte Carlo simulation, assessed the association between age categories and case classifications. The test indicated significant associations (p < 0.05), with an estimated p-value of 0.0161 from Monte Carlo simulation, confirming the significance despite data limitations. These results suggest significant associations between age categories and case classifications, though substantially limited by the small sample size and zero counts in some cells.

**TABLE 1:** Age distribution of patients by injury classification (Palestine, 2024).

Age group	Primary	Secondary	Tertiary
0–0	0	0	7
1–9	4	1	2
10–19	1	0	4
20–29	10	0	1
30–39	15	3	11
40–49	3	1	5
50–59	4	1	1
60+	4	1	4

**FIGURE 2:**
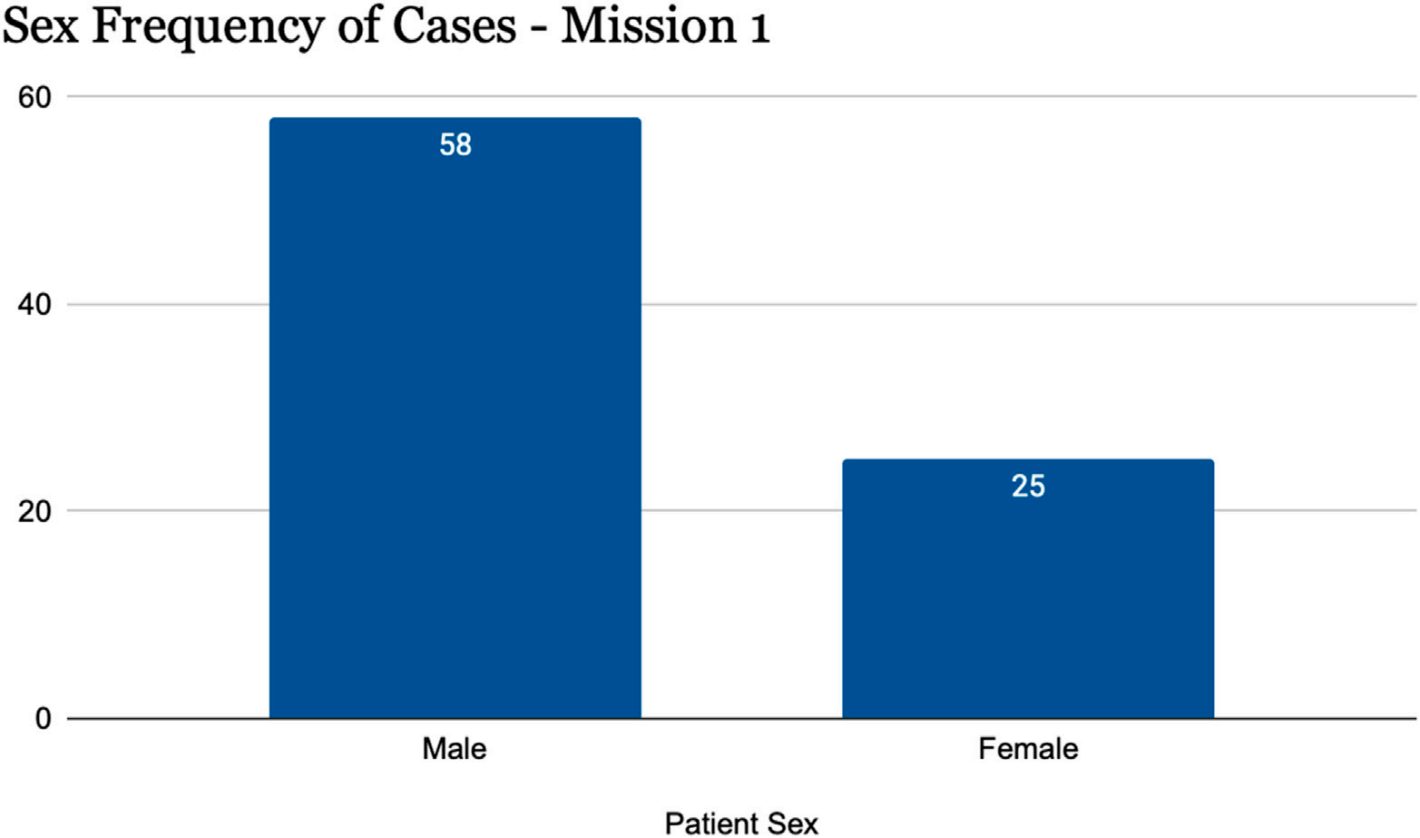
Trauma cases stratified by sex (Palestine, 2024).

The age distribution of cases showed a predominant skew towards male patients (Figure 2). 69.8% of the treated cases were male (n = 58), while 30.1% of the cases were female (n = 25).

### Surgical Intervention Linked Analysis

The operation data provide insights into the age distribution for various surgical procedures performed during the study period. Table 2 summarizes the mean, minimum, and maximum ages for each operation.

**TABLE 2:** Operation and age data (Palestine, 2024).

Operation name	Mean age	Min age	Max age
AVF thrombectomy	46	46	46
Above-the-knee Amputation	42	42	42
Amputation	37	37	37
Angioplasty	63	63	63
Angioplasty of Proximal SFA	76	76	76
Appendix Repair	7	7	7
Arteriovenous Fistula	79	79	79
Bilateral Intramedullary Nail	29	29	29
Bipolar hemiarthroplasty	65	65	65
Cast Placement and Examination	17.34	0.02	39
Catheterization of central venous occlusion of left Upper limb	48	48	48
Closed Hip Reduction + Hip Spica	0.17	0.17	0.17
Colostomy	8	8	8
Colostomy and ileostomy	0.1	0.1	0.1
Compartment Syndrome Fasciotomy	30	30	30
Compartment Syndrome Fasciotomy: Wash + Closure	30	30	30
Dressing under General Anesthesia (DUGA)	36.8	5	72
External Fixator Installation	31.62	9	55
External Fixator Removal	33.83	15	52
Foreign Body Removal	25.8	8	43
Herniotomy	0.33	0.33	0.33
Left brachiocephalic AVF	16	16	16
Nail Installation	26.8	9	37
Plate Insertion and Traction	41.93	9	85
Plate Removal	32	32	32
Skin Grafting	37	37	37
Tendon Repair	67	67	67
Wound Dressing	44.5	33	65
Duodenoduodenostomy	0.1	0.1	0.1
Pyloromyotomy	0.1	0.1	0.1

**FIGURE 3:**
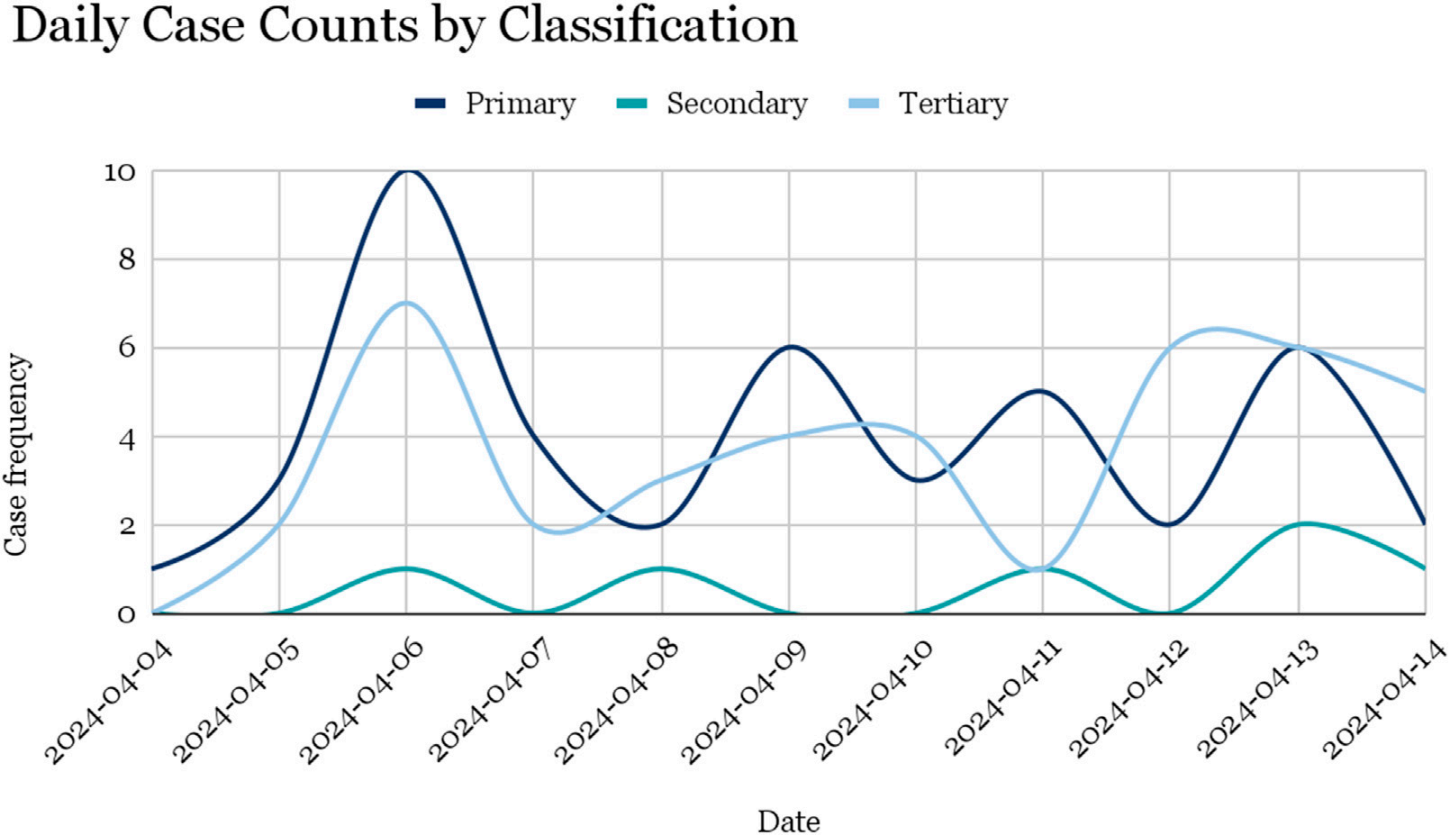
Daily trauma case counts by injury classification (Palestine, 2024).

The operation and age data reveal several trends (Figure 3). The youngest patients, with a mean age close to zero, underwent procedures such as closed hip reduction + hip spica, colostomy and ileostomy, and duodenoduodenostomy. Procedures like arteriovenous fistula and angioplasty of proximal SFA were performed on older patients, with mean ages of 79 and 76, respectively. The mean age for amputations was 37. Despite the results suggesting that observed trends in injury classifications were not statistically significant, it is clear through the qualitative assessment of data and operational subanalysis that specific age groups were more likely to be affected by different types of injuries.

The daily case counts by classification provide valuable insights into the temporal patterns of injuries treated during the conflict (Table 3). Several important trends are observed:

1. High Variability in Primary Injuries: The number of primary injuries fluctuated substantially from day to day, with the highest number of cases (10) occurring on April 6. This variability suggests that primary injuries, often resulting from direct conflict events, were influenced by the intensity and occurrence of hostilities on specific days.
2. Consistency in Secondary Injuries: Secondary injuries were consistently low throughout the study period, with most days reporting zero or one case. This consistency indicates that secondary injuries, which are indirectly related to the conflict, did not exhibit the same day-to-day fluctuations as primary injuries.
3. Tertiary Injuries and Healthcare Needs: Tertiary injuries showed notable numbers on several days, particularly on April 6 (7 cases) and April 13 (6 cases). These cases showcase the burden of ongoing healthcare needs unrelated to the conflict but requiring medical attention, such as chronic conditions and medical interventions necessary during the conflict period.
4. Impact of High-Casualty Days: Days with a high number of primary injuries, such as April 6 and April 13, likely placed a substantial strain on medical resources and healthcare personnel.

**TABLE 3:** Daily case counts by classification (Palestine, 2024).

Date	Primary	Secondary	Tertiary
2024-04-04	1	0	0
2024-04-05	3	0	2
2024-04-06	10	1	7
2024-04-07	4	0	2
2024-04-08	2	1	3
2024-04-09	6	0	4
2024-04-10	3	0	4
2024-04-11	5	1	1
2024-04-12	2	0	6
2024-04-13	6	2	6
2024-04-14	2	1	5

Primary injuries directly related to the war were the most frequent and severe, necessitating immediate and complex surgical interventions (Table 4). The most common surgeries performed for primary injuries included plate insertion and traction, totalling 12 cases. This was primarily used for the stabilization and fixation of fractures resulting from high-velocity impacts and explosive devices. Nail installation was required in 9 cases to treat long bone fractures, typically caused by blast trauma or gunshot wounds, necessitating internal stabilization. External fixator installation was performed in 5 cases to manage open fractures and severe soft tissue injuries, providing temporary stabilization of the limb. Wound care was necessary in 5 cases, addressing severe lacerations and soft tissue damage that required debridement and dressing changes. Foreign body removal was performed in 3 cases where shrapnel or other debris had embedded in the patients’ bodies due to explosions or gunfire.

**TABLE 4:** Surgery/case type frequency by case classification (Palestine, 2024).

Case classification	Operation name	Frequency
Primary	Plate Insertion and Traction	12
	Nail Installation	9
	External Fixator Installation	5
	Wound Care	5
	Foreign Body Removal	3
Secondary	Plate Insertion and Traction	2
	Appendix Repair	1
	External Fixator Installation	1
	External Fixator Removal	1
	Tendon Repair	1
Tertiary	Wound Care	8
	External Fixator Removal	4
	Cast Placement and Examination	2
	External Fixator Installation	2
	Congenital Malformation Corrections	2

Secondary injuries most often involved complications from previous trauma or accidents occurring amid the ongoing armed hostilities. The most common surgeries for secondary injuries were plate insertion and traction, necessary in 2 cases, addressing fractures from accidents or secondary trauma such as falls or structural collapses. One case involved an appendectomy. External fixator installation and removal were each performed once, and one case required tendon repair.

The most frequent surgeries in the category of tertiary injuries included wound care, performed in 8 cases. External fixator removal was performed in 4 cases, necessary for managing pre-existing orthopedic conditions that required follow-up care during the period of armed escalations. Cast placements were conducted in 2 cases, primarily for non-conflict-related fractures needing immobilization and follow-up. External fixators were installed in 2 instances, and there were 2 cases of congenital malformation corrections, such as pyloric stenosis and multiple duodenal atresia.

## Discussion

### Analysis of Demographic Trends and Injury Types

The data collected by FAJR during its emergency medical mission trip to Gaza serves as a snapshot dataset for the analysis of trauma cases at European Gaza Hospital (EGH). While this data only contains information from EGH, it serves as a useful tool to understand the general orthopedic surgical needs of the population of Khan Yunis and neighboring Rafah in the weeks immediately preceding the Israeli military ground offensive of Rafah in May 2024. Due to frequent instances of military raid, aerial bombardment, and destruction of healthcare facilities, EGH at the time in April 2024, and as of April 2025, remains one of the only free standing partially operational hospitals in the southern parts of the Gaza Strip equipped to handle such complex injuries (alongside Nasser Medical Complex and Al-Aqsa Martyrs’ Hospital). The analysis reveals that primary injuries, including blast injuries, gunshot wounds, and shrapnel injuries, predominantly affect middle-aged adults (30–39 years).

Several factors contribute to the higher incidence of primary injuries among adults, though children have been reported to also constitute a high proportion of conflict-related blast injuries [26]. One reason why an adult middle-aged demographic is more affected in the case of Gaza is that they are often more involved in high-risk occupations such as medical rescue workers, first responders, and essential service providers, all of which have been subject to frequent aerial-based and live-fire targeting, resulting in severe injuries that require immediate surgical intervention [27]. Economic and livelihood sustaining activities, such as scouring for food, water, essential medicines, or wood for fire can result in adults, particularly men, trekking into “red zones” that are marked for destruction [28, 29]. Furthermore, medical evacuations for middle-aged adults out of Gaza remains notoriously difficult, with a prioritization of children and elderly individuals prior to the Rafah ground invasion [30].

A lower frequency of children appearing in the primary data may be misleading, as children, women, and the elderly together constitute a majority of the current reported deaths and estimates of the toll, as well as those of direct traumatic injuries [9, 31]. Several factors contribute to the potentially higher lethality of direct injuries among children during armed conflicts [26, 32, 33]. Children’s lower physical resilience, smaller body size, thinner skin and less protective muscle mass make them susceptible to severe trauma wounds and subsequent mortality [34]. They are also more prone to hemodynamic instability, airway obstruction, and thermoregulation issues, which can complicate their recovery efforts [35–37].

Additionally, the chaotic environment of a war zone often impedes the prompt transportation of injured individuals to medical facilities, even more-so in Gaza [38, 39]. Children, in particular, may be trapped in unsafe conditions longer, either due to their dependence on adults for evacuation or because they are harder to identify and evacuate during rescue operations due to rubble and debris. Delays in accessing advanced medical care, such as surgical interventions, intensive care, and specialized pediatric care, increase the risk of mortality and severe morbidity among injured children [40]. Another factor to consider is that pediatric care requires specialized equipment and trained personnel which have been severely limited in Gaza due to the destruction of pediatric and neonatal facilities, and abduction and killings of specialists [41]. This shortage of pediatric surgeons, pediatric intensivists, and appropriate medical supplies, as documented by FAJR, further exacerbates the risk to injured children. The lack of suitable care for children in the immediate aftermath of injury can lead to higher fatality rates and severe long-term disabilities [42]. This disparity can contribute to an underrepresentation of pediatric cases in hospital records, as children who die before reaching medical facilities or being processed for surgical care are not documented in institutional data.

### Considering Secondary Injuries

The lower incidence rates of secondary injuries can be attributed to several factors. First, the immediate, life-threatening nature of primary injuries demands prioritization of medical resources and attention, overshadowing the treatment of secondary injuries at EGH and in Gaza as a whole. Civilians may also feel a sense of burden on the severely stressed healthcare system in Gaza by presenting to the hospital with less severe, secondary injuries. Second, those who sustain severe primary injuries may have reduced long-term survival rates, leading to fewer secondary complications being recorded. This high mortality rate among those with primary injuries reduces the potential pool of patients who might later present with secondary injuries. Indirect casualties have historically made up a substantial number of war-related deaths in conflict zones like Iraq and Sudan, thus limiting the proportion of indirect injuries presenting to the hospital at times [43]. Furthermore, the classification criteria and cataloging of patient admission data in EGH likely influenced the observed incidence of secondary injuries, given the constraints of staff and intensity of the conflict.

### Impact on Civilian Burden and Healthcare Capacity

The results of this study provide a useful lens to look into the effects of the Gaza war on orthopedic injuries. The high incidence of primary injuries, particularly among middle-aged adults, underscores the direct and devastating effects of military operations on noncombatants, indicating an overwhelming civilian burden due to military activities (Table 1). The findings also highlight concerns about the capacity of Gaza’s healthcare system to manage the surge in primary war-related injuries. The healthcare infrastructure in Gaza, already strained by prolonged conflict and limited resources, faces significant challenges in addressing both primary and tertiary injuries, as evidenced by case volume on high intensity days (Table 4) [44]. The study’s results indicate a substantial number of tertiary cases burdening the trauma system, including chronic conditions and congenital issues that persist and have been under-treated amid the armed conflict [45]. Tertiary injuries, including chronic conditions and congenital issues, demand long-term medical care and resources. The presence of congenital issues, potentially exacerbated by the unfavorable living conditions and environmental stressors in Gaza, further strains the healthcare infrastructure [46]. This demonstrated increased burden of civilian injuries provides a further snapshot into investigations and examinations of the scope of Israeli military operations in Gaza.

### Broader Implications and Recommendations for Medical Response

The findings of this study have implications for future medical interventions in conflict zones like Gaza. The high frequency of orthopedic surgeries among primary injury cases necessitates humanitarian actors focus on a robust trauma care strategy that includes immediate surgical interventions and long-term rehabilitation. This strategy should encompass a comprehensive trauma care protocol tailored to conflict environments, emphasizing rapid response capabilities and sustained care for complex injuries. The necessity of advanced orthopedic surgical supplies, such as external fixators, plates, and nails, along with the presence of skilled orthopedic surgeons, cannot be overstated. Medical missions should prioritize the availability of these resources to ensure that life-saving procedures can be performed promptly [47]. While in Gaza there are punitive blockades and restraints on the entry of goods and services, other conflict zones may not present the same challenges, and humanitarian medical groups should utilize such openings to develop capacity. Furthermore, the integration of rehabilitation services is crucial to address long-term disability and promote recovery, enabling individuals to regain functionality and improve their quality of life.

The study also reiterates the necessity of managing chronic conditions exacerbated by the conflict. Developing integrated healthcare programs that address both acute trauma and chronic orthopedic conditions, such as degenerative joint diseases and complications from previous injuries, can aid in efforts to provide continuous and comprehensive care [48, 49]. For instance, managing conditions like osteoarthritis, which may worsen due to lack of mobility and access to care, requires a consistent supply of medications, monitoring equipment, and physical therapy resources [50–52]. Pediatric care, given the noted vulnerability of children in conflict zones, necessitates the inclusion of pediatric orthopedic surgeons and appropriate medical supplies tailored to their unique needs. Ensuring the availability of pediatric ICU beds, ventilators, and specialized surgical instruments is essential. Pediatric care must extend beyond immediate surgical interventions to encompass long-term follow-up and support for congenital malformations and other chronic pediatric orthopedic conditions. For example, managing congenital conditions like clubfoot or developmental dysplasia of the hip (DDH) requires early intervention and sustained follow-up to prevent long-term disability. The integration of these specialized services into medical missions ensures that the healthcare needs of both adult and pediatric populations are met comprehensively, addressing the immediate and long-term impacts of conflict-related injuries.

To enhance the effectiveness of medical missions and healthcare responses, several recommendations can be made:

1. Increase Orthopedic Surgical Capacity: Ensure the availability of advanced orthopedic surgical supplies and expertise to manage the high incidence of severe fractures and soft tissue injuries.
2. Integrate Chronic Disease Management: Develop integrated healthcare programs that address both acute trauma and chronic conditions, providing holistic care to the population.
3. Prioritize Pediatric Care: Maintain specialized pediatric surgical expertise and resources to manage congenital malformations and other pediatric conditions effectively.
4. Improve Data Collection and Analysis: Adopt standardized data collection methods and electronic health records to enhance the accuracy and reliability of trauma data, facilitating better analysis and informing future medical interventions and resource allocation.

### Limitations and Future Research

While this study provides valuable insights into trauma cases in the Gaza war, several limitations should be acknowledged. The data collection was limited to the European Hospital in Khan Yunis in April 2024, which may not be representative of the broader Palestinian population’s medical needs. It provides a snapshot to look into the orthopedic surgical needs and interventions conducted prior to the Israeli military ground offensive in Rafah, and to the needs of those sheltering in the vicinity of EGH and the Al-Fukhari neighborhood. Additionally, the chaotic and resource-constrained environment of the conflict zone may have influenced the accuracy and completeness of the data. Potential biases, such as the prioritization of certain injury types due to the immediate medical mission’s focus, may also affect the findings. Future research should aim to include data from multiple healthcare facilities as well as a standardized collection method across NGOs and local medical teams to increase data points.

### Conclusion

This study demonstrates that the ongoing Gaza war continues to inflict severe burdens and traumatic injuries on the civilian population, including middle-aged adults who are often subjected to direct harm while fulfilling essential responsibilities or attempting to secure basic necessities. The role of primary injuries as dominant contributors to the trauma caseload underscores a harsh reality that civilian populations bear substantial risks in wartime environments, despite international frameworks intended to protect them from direct harm. Secondary and tertiary injuries showcase the complexity of morbidity causes and surgical intervention needs in conflict zones, where existing medical conditions and chronic illnesses are frequently worsened by interruptions to hospital functions and long-term care plans. These findings add another kernel to the documentation of a healthcare system, severely constrained by the blockade, infrastructure destruction, and disruptions to clinical workflows.

In the face of these considerable challenges, humanitarian organizations and medical NGOs have emerged as important forces for both immediate clinical intervention and data collection, though many of these efforts are still un-sustainable. Their activities can extend beyond traditional care delivery by systematically documenting injury patterns, treatment outcomes, and supply needs under conditions that often threaten their own safety. The ongoing targeting of journalists and healthcare professionals adds another layer of relevance and duty, compelling many of these organizations to serve as *de facto* record-keepers amid a climate of surveillance and restricted media access [53]. The phenomenon highlights a larger ethical tension where humanitarian actors strive to remain non-partisan while inevitably confronting the root causes of structural violence. Their documentation efforts can conflict with the external perception of neutrality, particularly when the blockade and ongoing hostilities hamper the delivery of life-saving supplies and obstruct the entry of specialized medical teams. Nevertheless, precise and credible data remain indispensable for advancing evidence-based interventions that address both urgent trauma care and longer-term rehabilitation.

The moral responsibilities carried by these NGOs reach into domains that carry beyond the delivery of medical aid or a specific surgical intervention. When reliable information on civilian harm is scarce, as it often is in Gaza due to technological blackouts and targeting of critical infrastructure, the systematic reporting of casualties, patient demographics, and surgical outcomes becomes a foundational aspect of public health advocacy. The findings outlined in this paper reveal it may be able to inform humanitarian policy or the goals of humanitarian actors, direct resources for reconstruction plans to the most acutely affected groups, and encourage further ethical deliberations. By shedding light on the extent of trauma among civilians, these organizations can shed light on the cumulative human cost of the blockade and bombardments that shape daily life in Gaza. Their work, therefore, represents an important intervention that challenges the notion of conflict as an unquantifiable tragedy, giving voice to populations that might otherwise remain invisible. NGOs have an ability to fulfill a vital role in the larger collective effort to reduce civilian suffering, shape international responses, and advocate for future measures that diminish harm, uphold medical neutrality, and respect some of the fundamental principles of humanitarianism, or consider reshaping them.
